# A novel and simple method for generation of human dendritic cells from unfractionated peripheral blood mononuclear cells within 2 days: its application for induction of HIV-1-reactive CD4^+^ T cells in the hu-PBL SCID mice

**DOI:** 10.3389/fmicb.2013.00292

**Published:** 2013-09-27

**Authors:** Akira Kodama, Reiko Tanaka, Mineki Saito, Aftab A. Ansari, Yuetsu Tanaka

**Affiliations:** ^1^Department of Immunology, Graduate School of Medicine, University of the RyukyusOkinawa, Japan; ^2^Department of Microbiology, Kawasaki Medical SchoolKurashiki, Japan; ^3^Department of Pathology, Emory University School of MedicineAtlanta, GA, USA

**Keywords:** dendritic cell, short-term culture, Th1-inducing DCs, anti-HIV-1 T cell response, hu-PBL-SCID

## Abstract

Because dendritic cells (DCs) play a critical role in the regulation of adaptive immune responses, they have been ideal candidates for cell-based immunotherapy of cancers and infections in humans. Generally, monocyte-derived DCs (MDDCs) were generated from purified monocytes by multiple steps of time-consuming physical manipulations for an extended period cultivation. In this study, we developed a novel, simple and rapid method for the generation of type-1 helper T cell (Th1)-stimulating human DCs directly from bulk peripheral blood mononuclear cells (PBMCs). PBMCs were cultivated in the presence of 20 ng/ml of granulocyte-macrophage colony-stimulating factor, 20 ng/ml of interleukin-4 (IL-4) and 1,000 U/ml of interferon-β for 24 h followed by 24 h maturation with a cytokine cocktail containing 10 ng/ml of tumor necrosis factor-α (TNF-α), 10 ng/ml of IL-1β and 1 μg/ml of prostaglandin E2. The phenotype and biological activity of these new DCs for induction of allogeneic T cell proliferation and cytokine production were comparable to those of the MDDCs. Importantly, these new DCs pulsed with inactivated HIV-1 could generated HIV-1-reactive CD4^+^ T cell responses in humanized mice reconstituted with autologous PBMCs from HIV-1-negative donors. This simple and quick method for generation of functional DCs will be useful for future studies on DC-mediated immunotherapies.

## INTRODUCTION

Dendritic cells (DCs) are professional antigen-presenting cells (APCs) which play a critical role in the regulation of the adaptive immune response through activation and polarization of naive T cells (Banchereau et al., 2000). Since small numbers of activated DCs are highly efficient in generating immune responses against infections and cancers (Moll and Berberich, 2001; Steinman and Banchereau, 2007), the DC therapy represents a new and promising immunotherapeutic approach for treatment of advanced cancers as well as for prevention of infectious diseases. Indeed, the current clinical trials with *ex vivo*-generated DCs (so-called DC vaccine) will yield precious information regarding their potentials as vectors for immunotherapy (Gilboa, 2007; Connolly et al., 2008; Ezzelarab and Thomson, 2011). However, the general protocols to generate DCs are complicated and time consuming. Moreover, since different *ex vivo* DC generation methods affect the DC phenotype and function (Kalantari et al., 2011), it is critical to choose appropriate method for generating functional DCs. In general, the DC precursor monocytes are purified from PBMCs by adherence (Jonuleit et al., 2001), elutriation (Berger et al., 2005) or positive or negative selection using immunomagnetic beads (Babatz et al., 2003). These enriched monocytes are then induced to differentiate into DCs by 5 days-*in vitro* cultivation in medium supplemented with granulocyte macrophage colony-stimulating factor (GM-CSF) and interleukin (IL)-4 followed by a 2-days-maturation procedure (Sallusto and Lanzavecchia, 1994; Gilboa, 2007; Dauer et al., 2008). However, a lines of evidence are increasing that mature monocyte-derived DCs can be generated even after short-term cell culture for 2–3 days (Dauer et al., 2003a, b; Jarnjak-Jankovic et al., 2007; Zhang et al., 2008; Tawab et al., 2009).

In this study, in an attempt to simplify the methods currently being used for optimal DC generation and to develop a standardized method of preparing effective myeloid DC vaccine for immunotherapies, we explored the efficacy of using unfractionated PBMCs as a source of DC precursors and short-term *in vitro* cell culture just for 2 days.

## MATERIALS AND METHODS

### REAGENTS

The media used were RPMI 1640 medium (Sigma, St. Louis, MO, USA) supplemented with 10% fetal calf serum (FCS; Sigma, St. Louis, MO, USA), 100 U of penicillin per ml, and 100 μg of streptomycin per ml (hereafter called RPMI medium) and Iscove’s modified Dulbecco’s medium (Lifetechnologies, Grand Island, NY, USA) supplemented with 10% FCS with the same antibiotics (hereafter called Iscove’s medium). Aldrithiol-2 (AT-2) and low-endotoxin bovine serum albumin (BSA) was purchased from Sigma (St. Louis, MO, USA). The recombinant human cytokines used included IL-4, GM-CSF, TNF-α and IL-1β (PeproTech, London, UK). Enzyme-linked immunosorbent assay (ELISA) kits for the quantitation of human IFN-γ, human IL-4, human IL-10 and human IL-12 (detecting IL-12 p75 heterodimer) were purchased from Biolegend. The human monocyte negative isolation kits and the human T cell isolation kits were purchased from Invitrogen (Carlsbad, CA, USA). The human naive CD4^+^ T cell isolation kit was purchased from Miltenyi Biotec (Gladbach, Germany). The Vybrant CFDA SE Cell Tracer Kit was purchased from Invitrogen.

### GENERATION OF DCs

Human PBMCs were isolated from heparinized peripheral blood obtained from normal healthy adult volunteer donors by standard density gradient centrifugation. Cells at the interface were collected and washed three times in cold phosphate-buffered saline (PBS) containing 0.1% low-endotoxin BSA and 2 mM Na_2_EDTA. For select experiments, monocytes were purified from PBMCs using the CD14^+^ monocyte negative isolation kit (Invitrogen, Carlsbad, CA, USA). An aliquot of cells from each monocyte preparation was examined by flow cytometry and found to contain >90% CD14^+^ cells. To obtain immature MDDCs (iMDDCs), PBMCs (2.5 × 10^6^ cells/ml) or the purified monocytes (5 × 10^5^ cells/ml) were cultured in RPMI medium containing 20 ng/ml of human GM-CSF and 20 ng/ml of human IL-4 at 37°C in 24-well plates in a 5% CO_2_ humidified incubator for 5 days. In other experiments, iDCs were generated from either purified monocytes or whole PBMCs by cultivation in RPMI medium containing GM-CSF (20 ng/ml), IL-4 (20 ng/ml) and IFN-β (1,000 U/ml) for 1 day. These iDCs were matured by incubation in the presence of either 10 ng/ml of LPS (Sigma) or a cocktail containing 10 ng/ml of TNF-α, 10 ng/ml of IL-1β and 1 μg/ml of prostaglandin E2 (PGE2; TIP cocktail) for 1–2 days.

### FLOW CYTOMETRY

Aliquots of the cells to be analyzed were incubated in PBS containing 0.1% BSA and 0.1% sodium azide (FACS buffer) supplemented with 2 mg/ml normal human IgG on ice for 15 min to block Fc receptors. The cell suspension was then incubated with a predetermined optimal concentration of the appropriate fluorescent dye-labeled mAbs against human cell surface markers on ice for 30 min. The fluorescent dye-labeled monoclonal antibodies (mAbs) against human cell surface molecules used included anti-CD3, anti-CD4, anti-CD8, anti-CD14, CD20, anti-CD80, anti-HLA-DR, and isotype-matched control mAbs (Beckman Coulter, Fullerton, CA, USA), and anti-CD11c, anti-CD86, and anti-CD83 (BioLegend, San Diego, CA, USA). After washing with FACS buffer, cells were fixed in 1% paraformaldehyde (PFA) containing FACS buffer. The cells were then analyzed on FACS-Calibur flow cytometer with CellQuest software (BD Pharmingen, San Diego, CA, USA). Isotype-matched mAbs were utilized as controls to stain an aliquot of the cells to be analyzed for purposes of establishing gates and for determination of the frequency of positively stained cells.

### HIV-1 PREPARATION AND INACTIVATION

HIV-1_IIIB_ (virus that only use CXCR4 as chemokine co-receptor, termed X4) was harvested from Molt-4/IIIB cell cultures. Batches of each HIV-1 preparation were inactivated with Aldrithiol-2 (AT-2; Sigma) as described previously (Yoshida et al., 2003). AT-2 was removed by three successive ultrafiltration in PBS using 100-kDa-cut-off centrifugal filtration devices (Centriprep 100; Amicon, Beverly, MA, USA). Then AT-2–inactivated HIV-1 (iHIV) was purified by pelleting down the virus at 20,000 × *g* for 2 h three times in 0.1% BSA-PBS. The virus pellet was resuspended in 0.1% BSA-PBS, aliquoted, and stored at -80°C until use. The concentration of HIV-1 was estimated by measuring levels of HIV-1 p24 antigen with our in-house p24 ELISA kit (Tanaka et al., 2010). As previously described (Yoshida et al., 2003), activated human PBMCs incubated with an aliquot of 1 μg/ml of the AT-2-treated HIV-1 preparation failed to demonstrate the presence of any detectable infectious virions (data not shown).

### STIMULATION OF T CELLS

Enriched populations of naive CD4^+^ T cells and bulk T cells with >90% purity were isolated from normal human PBMCs by using appropriate negative cell isolation kits. These T cells (4 × 10^4^ cells/well) were first labeled with carboxy-fluorescein diacetate succinimidyl ester (CFSE) according to the manufacturer’s instructions (Invitrogen, Carlsbad, CA, USA), then co-cultured with allogeneic DCs at a T cells: DCs ratio of 50:1 in 100 μl of RPMI medium supplemented with 20 U/ml human IL-2 in 96-well, U-bottomed plates. Cell proliferation and cytokine production were determined on day 4.

### hu-PBL-SCID MICE

The BALB/c-rag2^-/-^γc^-/-^ mice lacking T cells, B cells and natural killer (NK) cells (Rag2^-/-^ mice; Traggiai et al., 2004) were used in this study. The mice were kept in the specific-pathogen-free and P3 animal facilities of the Laboratory Animal Center, University of the Ryukyus. The protocols for the care and use of mice engrafted with human PBMCs and autologous DCs sensitized with inactivated HIV-1 or ovalbumin (OVA) were approved by the committee on animal research of the University of the Ryukyus prior to initiation of the study. Matured DCs (5 × 10^5^ cells) pulsed with either AT-2-inactivated HIV-1 (40 ng of p24) or 100 μg of OVA in 100 μl of RPMI medium for 2 h at 37°C were mixed with autologous fresh PBMCs (3 × 10^6^ cells) in a final volume of 100 μl in serum-free RPMI medium, and the were directly injected into the spleen of Rag2 mice as previously described (Yoshida et al., 2003). One week later, the same number of DCs pulsed with the same antigens were inoculated again into the spleen. One week later, mice were sacrificed, blood was collected by cardiocentesis, and human CD4^+^ T cells were enriched from splenocytes using a human CD4^+^ T cell isolation kit according to the manufacturer’s instructions. For the measurement of antigen-specific human cellular immune responses, human CD4^+^ T cell (2 × 10^5^ cells) collected from the spleens of immunized Rag2^-/-^ mice were cultured for 2 days with autologous monocytes (2 × 10^5^ cells) in the presence or absence of inactivated HIV containing 40 ng/ml of p24 in 500 μl of RPMI medium supplemented with 20 U/ml of IL-2 in individual wells of a 48-well plate at 37°C. The concentration of human IFN-γ or IL-4 produced in the culture supernatants was determined with ELISA kits.

### STATISTICAL ANALYSIS

Data were analyzed by Student’s *t* test with the with Prism software (GraphPad Software Inc., San Diego, CA, USA). 

## RESULTS

### GENERATION OF MYELOID MATURE DCs DIRECTLY FROM PBMCs WITHIN 2 DAYS

In order to reduce the cost, labor and any loss of potential precursors from PBMCs, we have previously established a novel culture method for generating functional human DCs from unfractionated PBMC in which whole PBMCs were cultured in the presence of IL-4 and GM-CSF for 5 days followed by a 2-day maturation in media containing poly I:C and IL-1β (Kodama et al., 2010). However, there were considerable lot variations in commercial poly:IC in the DC-maturation activity (data not shown). Therefore, we tested a previously reported maturation cytokine cocktail containing TNF-α, IL-1β, IL-6 and PGE2 (Jonuleit et al., 1997). In a preliminary study, we found that IL-6 was not necessary to mature DCs from purified monocytes in the present cell culture conditions, probably due to the use of serum-containing media. Thus, we used a cytokine cocktail containing 10 ng/ml of TNF-α, 10 ng/ml of IL-1β and 1 μg/ml of PGE2 (hereafter called TIP cocktail) throughout the present study.

**FIGURE 1:**
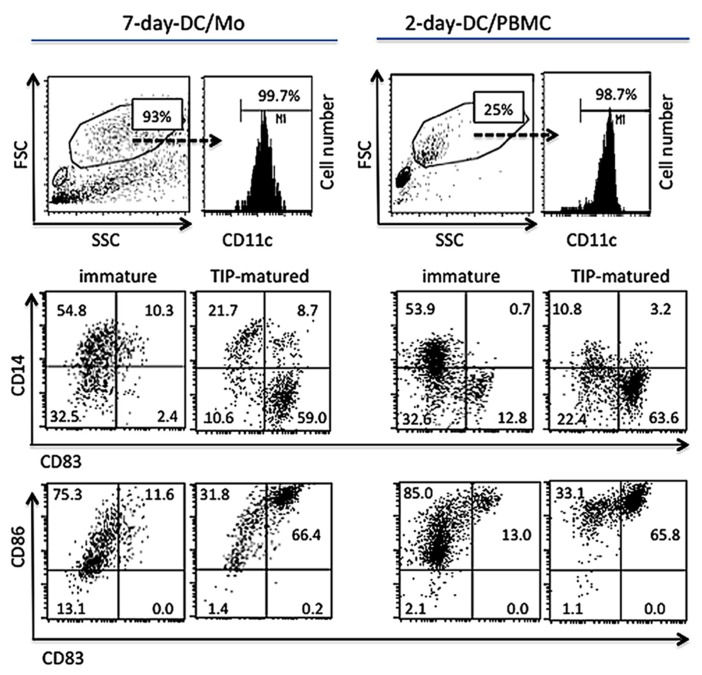
**Generation of functional human myeloid DCs directly from PBMCs *in vitro *within 2 days.** MDDCs generated purified monocytes for 7 days (7-day-DC/Mo) or whole PBMCs cultured in medium containing GM-CSF, IL-4 and IFN-β (2-day-DC/PBMC), either non-treated or treated with the maturation cocktail containing TNF-α, IL-1β and PGE2 (TIP) were examined for the expression of mature DC markers by a flow cytometry. Cells were stained with antibodies to CD11c, CD14, CD83 and CD86, and analyzed on a subpopulation gated for FSC^high^, SSC^high^ and CD11c^+^. The percentages of the positive cells in the gated population were shown in the dot plots. Data shown are representative of three independent experiments using blood from three different donors.

Based on our previous report that monocytes can be differentiated into mature DCs within 2 days (Zhang et al., 2008), we tested whether Th1-inducing DCs could be generated from unfractionated PBMCs. PBMCs (2.5 × 10^6^ cells/ml) were cultured in RPMI medium containing GM-CSF (20 ng/ml), IL-4 (20 ng/ml) and IFN-β (1,000 U/ml) for 1 day followed by additional 1 day cultivation in the presence or absence of the TIP cocktail. The phenotypes of CD11c^+^ large cells in these 2-day PBMC cultures were compared with those of MDDCs derived from purified monocyte for 7 days (7-day-DC/Mo; **Figure 1**). The proportion of FSC^high^ and SSC^high^ cells in the 2-day-DC/PBMC culture was 20~25% of total viable cells depending on donors and these cells expressed CD11c (data not shown). After maturation with the TIP cocktail, similar to the 7-day-DC/Mo, the large CD11c^+^ cells in the 2-day PBMC cultures became CD14^low^, CD86^high^ and CD83^ high^, a typical marker of matured myeloid DCs (Ohshima et al., 1997). The other viable cell populations in the 2-day PBMC cultures were CD3^+^ T cells (54.0~59.2%), CD56^+^ NK cells (8.4~9.3%) and CD19^+^ B cells (6.5~8.6%; *n* = 3). These data showed that the present culture method was applicable to generate myeloid mature DCs from bulk PBMCs within 2 days (2-day-DC/PBMC).

**FIGURE 2:**
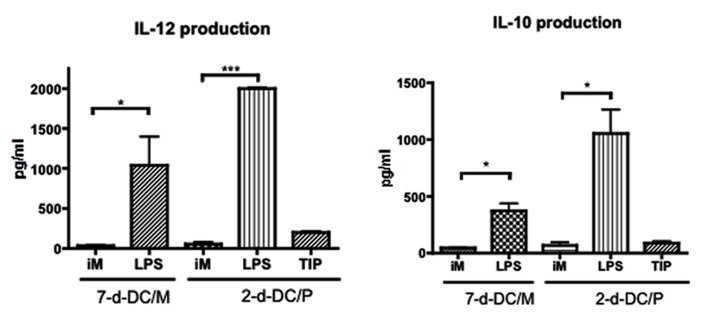
**Production of cytokines by the 2-day-DC/PBMC.** The levels of IL-12 (bioactive p70 heterodimer) and IL-10 in the supernatants from MDDCs (7-d-DC/M) or 2-day-DC/PBMC (2-d-DC/P) either non-matured (iM) or matured with LPS or the TIP cocktail were determined by ELISA. Data are mean ± SD of triplicate cultures. *P < 0.05, ***P < 0.001. Data shown are representative of three independent experiments using blood from two different donors.

**FIGURE 3:**
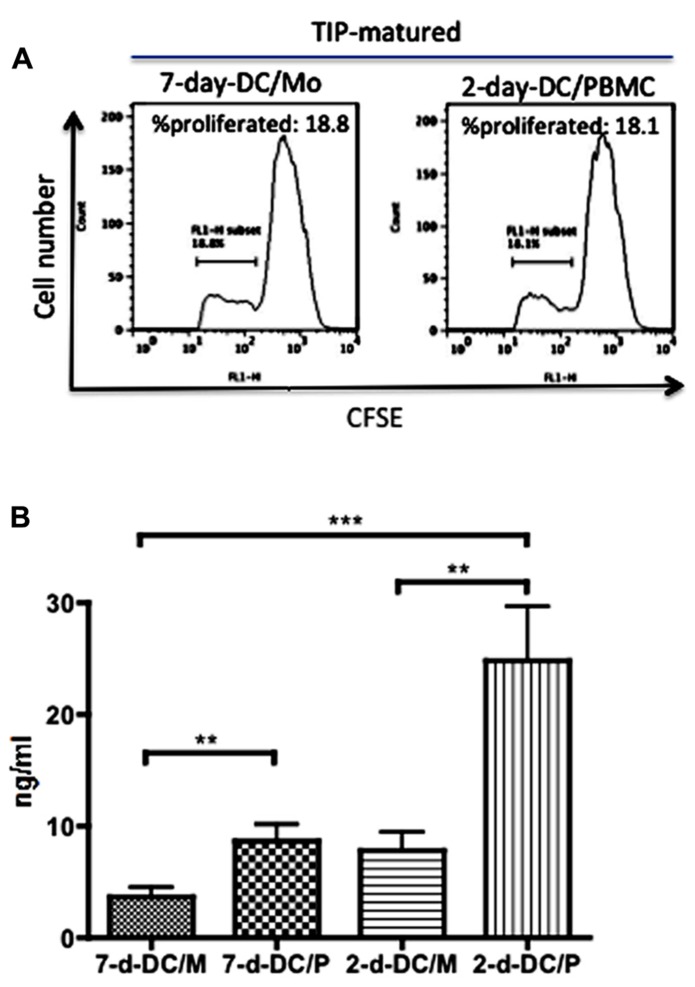
**High levels of production of IFN-γ by allogeneic T cells stimulated with 2-day-DCs generated from PBMCs.** Matured MDDCs (7-day-DC/Mo) and 2-day-DCs generated from PBMCs (2-day-DC/PBMC) were co-cultured with CFSE-labeled allogeneic CD4^+^ T cells at DC to T cell ratio of 1:50 for 4 days. The cell number of DCs in 2-day-DC/PBMC was manually counted using a Burker-Turk hemo-cytometer in which only large cells were counted. **(A)** Percentages of proliferated cells were examined by flow cytometry. **(B)** The levels of IFN-γ produced in the supernatants were quantitated by ELISA. In addition to the DCs shown in **(A)**, MDDCs generated from PBMCs for 7 days (7-d-DC/P) and 2-day-DCs generated from purified monocytes (2-d-DC/M) were also tested. Data shown are representative of three independent experiments using blood from two different donors.

Then we tested cytokine production by these 2-day-DC/PBMC. Interestingly, in contrast to the DCs matured in the presence of LPS, the production of IL-12 and IL-10 by the TIP matured 2-day-DC/P was minimum (**Figure 2**). To investigate whether the 2-day-DC/PBMC were immunologically functional, we examined their ability to stimulate allogeneic T cell proliferation. Like the MDDCs (7-day-DC/Mo), the 2-day-DC/PBMC could stimulate allogeneic T cell proliferation (**Figure 3A**). Then we quantitated the levels of IFN-γ and IL-4 in the culture supernatants from allogeneic CD4^+^ T cells co-cultured with various DCs. As shown in **Figure 3B**, among the four DC preparations including the 7-day-DC/Mo, 7-day-DCs from PBMCs (7-day-DC/PBMC), 2-day-DCs from monocytes (2-day-DC/Mo) and 2-day-DC/PBMC, the 2-day-DC/PBMC were most potent in induction of IFN-γ production. The bulk 2-day-DC/PBMC alone did not produce detectable IFN-γ (<20 pg/ml) in the present culture conditions (data not shown). The levels of IL-4 and IL-10 were below detection (<5 pg/ml) in all the samples tested (data not shown). These results indicated that the 2-day-DC/PBMC had a potential to induce Th1 response.

### INDUCTION OF HIV-1-REACTIVE HUMAN CD4^+^ T CELL RESPONSES IN hu-PBL-SCID MICE

**FIGURE 4:**
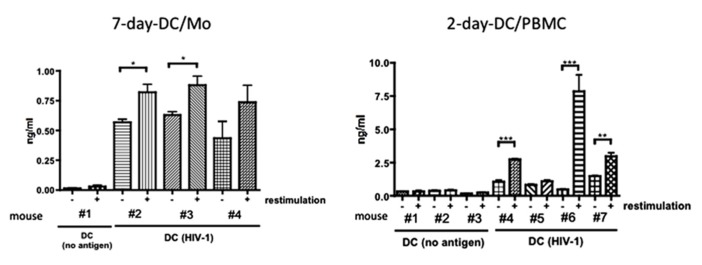
**Functional activity of the 2-days-DCs in hu-PBL-SCID mice.** Fresh autologous PBMCs from normal human donors were transferred into the Rag2^-/-^ mouse spleen together with autologous mature MDDCs (7-day-DC/Mo) or 2-days-PBMC-derived DCs (2-day-D/PBMC) pulsed with no antigen (no antigen) or AT-2-inactivated HIV-1 (40 ng of p24). On day 7 after the first transplantation, these mice were received an *intra splenic* booster injection with similarly prepared DCs. Seven days after the booster injection, mice were sacrificed and human CD4^+^ T cells were purified from splenocytes. These CD4^+^ T cells were co-cultured with autologous APCs (adherent PBMCs) in the presence or absence of antigens (restimulation) for 2 days at 37°C. IFN-γ levels produced in the culture supernatants were measured by ELISA. Data show mean ± SD of triplicate cultures. *P < 0.05, **P < 0.01, ***P < 0.001. Data shown are representative of three independent experiments using blood from two different donors.

Finally, we examined whether the short-term generated 2-day-DC/PBMC could induce HIV-1-reactive immune responses *in vivo* in comparison to MDDCs (7-day-DC/Mo) using our hu-PBL-SCID mice model (Yoshida et al., 2003). SCID mice were *intra-splenically* transplanted with DCs loaded with AT-2-inactivated HIV-1 together with autologous fresh PBMCs. On day 7, these mice were received an *intra-splenic* booster injection with similarly prepared antigen-pulsed DCs. Seven days after the booster injection, mice were sacrificed and examined for antigen-specific human immune responses. **Figure 4** showed that after* in vitro* re-stimulation with autologous APCs pulsed with inactivated HIV-1, enriched human CD4^+^ T cells from two out of three mice immunized with MDDCs (7-day-DC/Mo) pulsed with HIV-1 and those from three out of four mice immunized with 2-day-DC/PBMC pulsed with HIV-1 produced IFN-γ in antigen-dependent way, indicating that the 2-day-DC/PBMC could induce HIV-1 antigen-reactive human T responses *in vivo* as potent as MDDCs. In the re-stimulated culture supernatants, no IL-4 or IL-10 was detected (<5 pg/ml) using ELISA (data not shown). In addition, no detectable antibodies against HIV-1 were detected as determined by using a commercial Western blot assay kit in plasma samples from all the DCs-HIV-1-immunized mice (data not shown).

Altogether, these results demonstrated that human myeloid DCs directly generated from PBMCs by the present short-term cultivation method were potent in induction of functional Th1 responses both *in vitro* and *in vivo*.

## DISCUSSION

In the present study, we have developed a novel, simple and rapid protocol for generating Th1-stimulating human myeloid DCs directly from unfractionated PBMCs. These 2-day-DC/PBMC were potent in both stimulating allogeneic T cells *in vitro* and inducing HIV-1-reactive Th1 responses in hu-PBL-SCID mice. The use of whole PBMCs as DC precursors might reduce any loss of monocytes in the step of purification by adherence (Jonuleit et al., 2001), elutriation (Berger et al., 2005) or positive or negative selection using immunomagnetic beads (Babatz et al., 2003). One possible concern on using whole PBMCs was that the non-monocyte cells, such as T, B or NK cells, in the PBMCs might interfere with differentiation and function of DCs. However, in the present study there was no obvious difference in DC maturation and function between in PBMC and purified monocyte cultures.

For the final maturation, we used a cytokine cocktail containing TNF-α and IL-1β and PGE2 (TIP coctail). Simultaneous use of these three reagents in TIP was essential for maturation of DCs since use of the reagents either in single or in two combinations failed to mature DCs (data not shown). In general, IL-6 that is included in the maturation cytokine cocktail TNF-α and IL-1β and PGE2 to mature DCs was not necessary in the present culture conditions. The reason remains to be studied, but it is possible that IL-6 is required in serum-free culture conditions. The present 2-day-DC/PBMC matured by TIP produced lower IL-12 than those matured by LPS. Low levels production of IL-12 might be ascribed to the use of PGE2 that inhibits bioactive IL-12 heterodimer production (Kalinski et al., 2001; Kalim and Groettrup, 2013). Despite of the low level production of IL-12, the TIP-matured 2-day-DC/PBMC were potent in stimulating IFN-γ, but not IL-4 or IL-10, production by allogeneic T cells. The reason for higher potentials of 2-day-DC/PBMC to induce Th1 cells than MDDCs remains to be clarified. It is speculated that natural DCs contained in the 2-day-PBMC-derived DCs might enhance the activation. Indeed, 2-day-DC/PBMC culture generated from CD14^+^ cell-depleted PBMCs were able to stimulate allogeneic CD4^+^ T cells to a lesser extent (data not shown). However, we cannot clearly determine if the stimulation was mediated by remaining monocytes. Further study is required to solve this issue. Importantly, as the previous study (Yoshida et al., 2003), the present study showed the induction of primary HIV-1-specific human CD4^+^ T cell immune responses in hu-PBL-SCID mice by DC-based immunization, demonstrating that the present 2-day-PBMC-derived DCs might have a potential for clinical use in DC-based immunization in humans against HIV-1. It was of interest that the levels of IFN-γ production were higher in CD4^+^ T cells immunized with 2-day-DC/PBMC than those immunized with 7-day-DC/Mo. It is possible that 2-day-DC/PBMC could live longer than 7-day-DC/Mo *in vivo *to stimulate antigen-specific CD4^+^ T cells. In addition, because myeloid DCs are susceptible to HIV-1 infection (Knight et al., 1990), the use of these IFN-β-treated DCs will be beneficial for HIV-1-infected individuals.

In conclusion, the present study provided a new method to generate functional human myeloid DCs directly from PBMCs in a short-term culture period. These DCs will be useful for studies exploring potentials of DC-based immunization for not only infectious diseases but also cancers *in vitro* and *in vivo*.

## Conflict of Interest Statement

The authors declare that the research was conducted in the absence of any commercial or financial relationships that could be construed as a potential conflict of interest.

## Author Contributions

Akira Kodama designed and performed the experiments, analyzed the data and wrote the paper. Reiko Tanaka and Mineki Saito performed the experiments, analyzed the data and wrote the paper. Aftab A. Ansari participated in the design of the study and helped to draft the manuscript. Yuetsu Tanaka designed and supervised the research, performed experiments and wrote the paper. All authors checked the final version of this manuscript.
